# Implications of the medication regimen complexity index score on hospital readmissions in elderly patients with heart failure: a retrospective cohort study

**DOI:** 10.1186/s12877-023-04062-2

**Published:** 2023-06-19

**Authors:** Asmaa Abdelbary, Rasha Kaddoura, Sara Al Balushi, Shiema Ahmed, Richard Galvez, Afif Ahmed, Abdulqadir J. Nashwan, Shaikha Alnaimi, Moza Al Hail, Salah Elbdri

**Affiliations:** 1 Pharmacy Department, Community and Home Health Services, Hamad Medical Corporation, Doha, Qatar; 2 Pharmacy Department, Heart Hospital, Hamad Medical Corporation, Doha, Qatar; 3 Pharmacy Department, Communicable Disease Center, Hamad Medical Corporation, Doha, Qatar; 4 Corporate Pharmacy Department, Hamad Medical Corporation, Doha, Qatar; 5 Nursing Department, Hamad Medical Corporation, P.O. Box 3050, Doha, Qatar; 6 Cardiology Department, Heart Hospital, Hamad Medical Corporation, Doha, Qatar

**Keywords:** Elderly, Heart failure, Medication regimen complexity, Polypharmacy

## Abstract

**Background:**

The likelihood of elderly patients with heart failure (HF) being readmitted to the hospital is higher if they have a higher medication regimen complexity index (MRCI) compared to those with a lower MRCI. The objective of this study was to investigate whether there is a correlation between the MRCI score and the frequency of hospital readmissions (30-day, 90-day, and 1-year) among elderly patients with HF.

**Methods:**

In this single-center retrospective cohort study, MRCI scores were calculated using a well-established tool. Patients were categorized into high (≥ 15) or low (< 15) MRCI score groups. The primary outcome examined the association between MRCI scores and 30-day hospital readmission rates. Secondary outcomes included the relationships between MRCI scores and 90-day readmission, one-year readmission, and mortality rates. Multivariate logistic regression was employed to assess the 30- and 90-day readmission rates, while Kaplan-Meier analysis was utilized to plot mortality.

**Results:**

A total of 150 patients were included. The mean MRCI score for all patients was 33.43. 90% of patients had a high score. There was no link between a high MCRI score and a high 30-day readmission rate (OR 1.02; 95% CI 0.99–1.05; p < 0.13). A high MCRI score was associated with an initial significant increase in the 90-day readmission rate (odd ratio, 1.03; 95% CI, 1.00-1.07; p < 0.022), but not after adjusting for independent factors (odd ratio, 0.99; 95% CI, 0.95–1.03; p < 0.487). There was no significant difference between high and low MRCI scores in their one-year readmission rate.

**Conclusion:**

The study’s results indicate that there is no correlation between a higher MRCI score and the rates of hospital readmission or mortality among elderly patients with HF. Therefore, it can be concluded that the medication regimen complexity index does not appear to be a significant predictor of hospital readmission or mortality in this population.

**Supplementary Information:**

The online version contains supplementary material available at 10.1186/s12877-023-04062-2.

## Introduction

Early readmissions are common among patients hospitalized with heart failure (HF), with majority of readmissions are due to causes other than HF [1]. After an index hospitalization with HF, 30- and 90-day readmissions for HF and all-cause mortality had increased [2]. The rates of HF rehospitalization were estimated as 18% and 40% and the rates of cumulative mortalitywere 13% and 20% at 3 and 12 months, respectively, in seven Middle Eastern countries including Qatar [3]. Risk factors for HF readmissions are multifactorial; with some deemed modifiable [4, 5]. Medications-related problems such as number of medications, are one of those risk factors which are associated with unplanned hospital readmissions in elderly HF patients [6, 7]. Polypharmacy, defined as taking five or more medications, is only one element of medication regimen complexity [8]. Other elements that can contribute to regimen complexity include the multiple characteristics of the prescribed regimen and its other related factors such as medication administration, number of different dosage forms, dosing frequencies, and special instructions for medication use. Consequently, patient’s adherence to medication intake can negatively be affected [9, 10]. The validated tool known as Medication Regimen Complexity Index (MRCI) comprises 65 items that evaluate the dosage forms, dosing frequencies, and supplementary instructions for each medication administered [11]. Several studies have suggested that the complexity of medication regimens and accompanying instructions can result in medication errors and unfavorable clinical outcomes. As a result, some researchers have proposed that MRCI may be a useful tool for predicting adverse clinical outcomes, surpassing the simple medication count [12]. The validity of the MRCI tool is deemed satisfactory, and its reliability in evaluating medication regimen complexity has been established in both heart failure (HF) and chronic obstructive pulmonary disease (COPD) [13, 14]. The current evidence on the impact of MRCI use on patients’ clinical outcomes is inconclusive and had shown inconsistent results [15]. Some studies had confirmed an association between MRCI and hospital readmissions [16–20], while other studies had failed to prove the same finding [21, 22]. In the State of Qatar, 31.4% of the population are elderly (i.e., age above 65 years old) as per the 2018 published statistics [23], suggesting higher risk of cardiovascular diseases occurring among Qatari older population [24]. Thus, the management of cardiac conditions, such as HF, can be more challenging to the health care system due to multiple co-morbidities, frailty, and polypharmacy [25–27]. The prevalence of polypharmacy and potentially inappropriate medications among Qatari elderly patients is high [28, 29]. A study that investigated the prevalence of polypharmacy in elderly patients with cardiovascular diseases, found that its prevalence rates ranged from 17.2 to 88.6% in Qatar and the Middle East and North Africa region [30]and it is negatively affecting our elderly population [32]. The aim of the present study is to determine the association between the MRCI scores and hospital readmission and mortality rates in elderly patients with HF.

## Methods

This is a single-center retrospective cohort study conducted at the Heart Hospital, a tertiary hospital affiliated with Hamad Medical Corporation in Qatar. The study site is an ambulatory advanced HF clinic, established in 2015, that provides specialized multidisciplinary care and follow-up for patients with HF. The protocol was approved by the corporation’s Medical Research Center (MRC-01-20-025) in September 2020. The patients’ data review and collection began in October 2020 and lasted for three months. Patients were identified and randomly selected from the clinic’s electronic database between January 1 and December 31, 2018, and were followed for up to 12 months. Patients were eligible if they were 65 years or older and were referred to the clinic after being discharged from the hospital with a diagnosis of decompensated or acute HF exacerbation. Exclusion criteria included emergency visits, admission not related to decompensated HF, discharge against medical advice, and when the medication list was inaccessible. The index discharge date, defined as the date of hospital discharge before the first visit to the HF clinic, was the starting point for the screening of the discharge medication list from electronic medical records. The MRCI score was manually calculated by independent investigators for the eligible patients using a validated tool which was published by George et al. in 2004 [11]. Every third patient was selected from the electronic database, until reaching the planned number of 150 patients. The tool has three sections (A, B and C). In Section A, higher weights are assigned to medications with inconvenient dosage forms or more difficult to administer (e.g., an oral tablet receives 1 point while a metered-dose inhaler receives 4 points). In Section B, medications that are administered more frequently or at more strict time intervals receive more points (e.g., “twice daily” receives 2 points, while “every 12 h” receives 2.5 points). Finally, in Section C, further points are assigned if the medication regimen indicates any additional instructions such as “break/crush tablet” (1 point) or “taper dose as directed” (2 points) [11]. Patients were stratified into patients with low (< 15) or high (≥ 15) MRCI score. The cut-off score of 15 was reported to be linked to hospital readmission [15, 16]. The primary outcome of this study is to determine the association between the MRCI score, and the hospital readmission rates in elderly HF patients. The 30-day readmission rate was counted from the index discharge date until hospital readmission. The secondary outcomes included MRCI association with 90-day readmission rate, number of readmissions and one-year mortality rate.

### Statistical analysis

A sample size of 150 patients was calculated based on a previous study in which the odds of having MRCI score of ≥ 15 was higher by 62% among patients who were readmitted to hospital within 30 days of discharge [18]. A cut-point of 15 for MRCI was used to dichotomize the score. Baseline characteristics of the participants were tabulated using proportions (percentages) for categorical variables and mean for continuous variables. The variables including etiology of HF, ejection fraction, diabetes mellitus, chronic kidney disease, Charlson score, and total number of medications were considered clinically important as per the literature review. Readmission rates (30-day and 90-day) were evaluated by purposeful model building of a multivariate logistic regression to adjust for the independent variables. The model was validated by correctly assessing classified predictions, ROC curve andHosmer-Lemeshow and Pearson Chi-square goodness of fit tests. The number of readmissions was evaluated by Poisson regression to adjust for the independent variables. Mortality was analyzed by chi-square test of independence. All tests were two-sided, with *B* set at 80% and a *P*‐value of < 0.05 to be considered significant. All statistical analysis was performed using Stata/SE 14.2. An online software calculator was used to determine the sample size with margin of error of 5% (significance level = 0.5) [32].

## Results

A total of 150 patients were included in this study. The mean age was 74.4 (standard deviation (SD) 6.29) years old, and 82 (54.67%) of the patients were males, 81 (54%) were Qatari and 69 (46%) were of other nationalities. The mean MRCI score of all patients was 33.43 (SD 15.47). Most of the patients (90%) had high MRCI (≥ 15) scores and 88% were using ≥ 10 medications. The baseline characteristics are described in Table 1. The 30- and 90-day re-admission rates in the high MRCI group were 14% (P = 0.120) and 15% (P = 0.109), respectively, as compared with no re-admissions in the low MRCI group at both follow-up periods. Mortality was not significantly higher among patients with high MRCI score (30% versus 13%, P = 0.182) (Table 2). The univariate analysis did not show a significant association between MRCI score and the 30-day re-admission rate (odds ratio, 1.02; 95% CI 0.99–1.05; P = 0.13) even after adjusting for all the independent factors (Table 3). Although the univariate analysis showed a significantly slight increase in the 90-day re-admission rate with high MRCI score (odds ratio, 1.03; 95% CI, 1.00–1.07; P = 0.022), there was no significant association after adjusting for the independent factors (Table 4). The number of re-admissions, using Poisson regression, was significantly higher in those with high MRCI score (odds ratio, 1.011; 95% CI, 1.001–1.018; P = 0.001), but not after adjusting for other factors (odds ratio, 1.006; 95% CI, 0.999–1.014; P = 0.093) (Table 5). Deaths during the follow-up period are plotted for both patients with low and high MRCI scores using Kaplan-Meier time-to-event curve in Supplementary Fig. 1. An exploratory analysis was conducted using MRCI score of 30.5 (the median) as the cut-off point to dichotomize the groups to low (N = 72) and high (N = 78) MRCI scores. Results were comparable with no significant differences between the exploratory cohorts.

**Table 1:** Baseline characteristics of patients

Characteristic	Overall	Low MRCI score N = 15	High MRCI score N = 135
Age, mean (SD)	74.4 (6.3)	73.2 (4.5)	74.5 (6.5)
Male, no. (%)	82 (54.7)	9 (60)	73 (54)
Qatari nationality, no. (%)	81(54)	4 (26.7)	77 (57)
Marital status, no. (%)			
Married	131 (87.3)	12 (80)	119 (88.2)
Divorced	1 (0.7)	0 (0)	1 (0.7)
Widowed	7 (4.7)	1 (6. 7)	6 (4.4)
Unknown	11 (7.3)	2 (13.3)	9 (6.7)
Smoking status, no. (%)			
Smoker	9 (6)	2 (13.3)	7 (5.2)
Ex-smoker	23 (15.3)	1 (6. 7)	22 (16.3)
Never	55 (36. 7)	5 (33.3)	50 (37)
Unknown	63 (42)	7 (46.7)	56 (41.5)
MRCI score, mean (SD)	33.43 (15.5)	11.9 (2.5)	35.81 (14.4)
HF etiology, no. (%)			
ICM	108 (72)	10 (66.7)	98 (72.6)
HF, no. (%)			
HFmEF	11 (7.3)	1 (6.7)	10 (7.4)
HFpEF	52 (34.7)	4 (26.7)	48 (35.6)
HFrEF	87 (58)	10 (66.7)	77 (57)
Co-morbidities, no. (%)			
Diabetes mellitus	124 (82.7)	10 (66.7)	114 (84.4)
Hypertension	139 (92.7)	14 (93. 3)	125 (92.6)
Respiratory disease	56 (37.3)	1 (6.7)	55 (40.7)
CKD or any kidney disease	80 (53.3)	7 (46.7)	73 (54.1)
On dialysis	7 (4.7)	1 (6.7)	6 (4.4)
Chronic liver disease	7 (4.7)	0 (0)	7 (5.2)
Cancer	18 (12)	3 (20)	15 (11.1)
Solid tumor	14 (9.3)	2 (13.3)	12 (8.9)
Leukemia	1 (0. 7)	0 (0)	1 (0.7)
Cerebrovascular disease	25 (16. 7)	0 (0)	25 (18.5)
Charlson Score, mean (SD)	7.95 (2.4)	6.8 (1.9)	8.1(2.5)
Total number of medications, no. (%)			
≥ 5	4 (2.7)	4 (26. 7)	0 (0)
≥ 7	14 (9.3)	8 (53.3)	6 (4.4)
≥ 10	132 (88)	3 (20)	129 (95.6)

CKD: Chronic kidney disease, HF: Heart failure, HFmEF: Heart failure with mid-range ejection fraction, HFpEF: Heart failure with preserved ejection fraction, HFrEF: Heart failure with reduced ejection fraction, ICM: Ischemic cardiomyopathy, MRCI: Medical regimen complexity index, SD: Standard deviation

**Table 2:** Outcomes according to MRCI score

Outcome, no. (%)	Low MRCI (n = 15)	High MRCI (n = 135)	P value
30-Day readmission	0 (0%)	19 (14%)	P = 0.120
90-Day readmission	0 (0%)	20 (15%)	P = 0.109
Mortality	2 (13%)	40 (30%)	P = 0.182

MRCI: Medical regimen index

**Table 3:** Univariate and multivariate analyses of 30-day re-admission

Characteristic	Unadjusted OR (95% CI)	P value	Adjusted OR (95% CI)	P value
MRCI score	1.02 (0.99–1.05)	0.13	1.01 (0.98–1.05)	0.430
HF etiology				
ICM	Ref			
NICM	1.22 (0.08–0.24)	0.71	0.92 (0.3–2.90)	0.882
HF				
HFmEF	Ref			
HFpEF	1.21 (0.23–6.42)	0.825	1.03(0.17–6.17)	0.942
HFrEF	0.33 (0.06–1.9)	0.216	0.26 (0.043–1.57)	0.142
Diabetes mellitus	1.9 (0.41 − 0.35)	0.409	1.28 (0.24–6.93)	0.744
CKD or any kidney disease	1.24 (0.47–3.27)	0.670	0.823 (0.22–3.08)	0.773
Charlson score	1.09 (0.9–1.32)	0.370	1.13 (0.87–1.48)	0.361

CI: confidence interval, CKD: Chronic kidney disease, HF: Heart failure, HFmEF: Heart failure with mid-range ejection fraction, HFpEF: Heart failure with preserved ejection fraction, HFrEF: Heart failure with reduced ejection fraction, ICM: Ischemic cardiomyopathy, NICM: Non ischemic cardiomyopathy, MRCI: Medical regimen complexity index, OR: odds ratio, SD: Standard deviation

**Table 4:** Univariate and multivariate analyses of 90-day re-admission

Characteristic	Unadjusted OR (95% CI)	P value	Adjusted OR (95% CI)	P value
MRCI score	1.03 (1.00–1.07)	0.022	1.03 (0.99–1.06)	0.103
HF etiology				
ICM	Ref			
NICM	1.88 (0.71–5.00)	0.204	2.24 (0.72–6.96)	0.162
HF				
HFmEF	Ref			
HFpEF	1.56 (0.17–14.10)	0.825	0.58 (0.05–6.46)	0.659
HFrEF	1.6 (0.19–13.65)	0.667	1.13 (0.122–10.41)	0.915
Diabetes mellitus	2.04 (0.44–9.38)	0.361	0.78 (0.14–4.38)	0.777
CKD or Any kidney disease	3 (1.03–8.74)	0.044	2.71 (0.69–10.63)	0.153
On dialysis	10.58 (2.17–51.62)	0.004	4.49 (0.55–36.87)	0.162
Charlson score	1.18 (0.98–1.43)	0.081	1.05 (0.75–1.46)	0.778

CI: confidence interval, CKD: Chronic kidney disease, HF: Heart failure, HFmEF: Heart failure with mid-range ejection fraction, HFpEF: Heart failure with preserved ejection fraction, HFrEF: Heart failure with reduced ejection fraction, ICM: Ischemic cardiomyopathy, MRCI: Medical regimen complexity index, NICM: Non ischemic cardiomyopathy, OR: odds ratio, SD: Standard Deviation

**Table 5:** Univariate and multivariate analyses for number of re-admissions

Characteristic	Unadjusted IRR (95% CI)	P value	Adjusted IRR (95% CI)	P value
Age	0.99 (0.98–1.07)	0.778	0.96 (0.87–1.06)	0.391
Male	0.72 (0.581–0.91)	0.004	0.734 (0.586–0.918)	0.007
Nationality				
Non-Qatari	Ref			
Qatari	2.909 (0.999–8.472)	0.050	3.56 (0.95–13.40)	0.060
MRCI score	1.011 (1.001–1.018)	0.001	1.006 (0.999–1.014)	0.093
HF etiology				
ICM	Ref			
NICM	0.929 (0.732–1.779)	0.541	0.849 (0.654–1.102)	0.219
HF				
HFmEF	Ref			
HFpEF	2.294 (1.726–3.051)	0.001	2.11 (1.504–2.972)	0.001
HFrEF	1.838 (1.455–2.323)	0.001	1.748 (1.333–2.292)	0.001
Diabetes mellitus	1.463 (1.141–1.876)	0.003	1.229 (0.980–1.541)	0.075
CKD or any kidney disease	1.126 (0.892–1.421)	0.318	1.008 (0.753–1.349)	0.957
Charlson score	1.045 (1.003–1.089)	0.037	1.020 (0.964–1.079)	0.500
Total number of medications				
≥ 5	0.521 (0.363–0.747)	0.001	0.797 (0.443–1.434)	0.450
≥ 7	0.625 (0.457–0.854)	0.003	0.792 (0.538–1.166)	0.237
≥ 10	Ref			

CKD: Chronic kidney disease, HF: Heart failure, HFmEF: Heart failure with mid-range ejection fraction, HFpEF: Heart failure with preserved ejection fraction, HFrEF: Heart failure with reduced ejection fraction, ICM: Ischemic cardiomyopathy, IRR: Incidence rate ratio, MRCI: Medical regimen complexity index, SD: Standard deviation

## Discussion

This study did not show an association between the MRCI score and hospital readmissions, total number of admissions or mortality. High MRCI scores in patients with chronic kidney disease and dialysis were initially associated with 90 days readmission using univariate analysis, but after using multivariate logistic regression, there was no significant association. The total number of readmissions within 12 months was initially significantly higher in those with high MRCI and elevated Charlson comorbidity index and polypharmacy compared to those with low MRCI, and it was more predominant among the Qatari population compared to other nationalities, but again this was non-significant after adjusting other variables. In our study, although the mortality rate was not statistically significantly higher in the high MRCI group, it may be of clinical significance, which may confirm the results of the previously published study that concluded higher survival rate in elderly patients with simplified regimen compared to those with complex regimen [33]. The previously published studies investigating MRCI had focused on elderly patients with multiple co-morbidities, polypharmacy, and subsequently higher medication regimen complexity [34, 35]. This was demonstrated in our population of this study and in similar studies done before, indicating that polypharmacy is prevalent in elderly population in Qatar [30, 31]. While hospital readmission was one of the most common health care outcomes studied in association with the MRCI score, data showed mixed results. Our finding is opposing the predictive association that was confirmed by some studies [16–20], but consistent with the results of other studies [21, 22]. For example, Yam and his colleagues concluded that medication modification upon hospital discharge for elderly HF patients usually results in subsequent increase in mean MRCI by 50% and it is harmless [22]. The lack of association between high MRCI scores and hospital re-admissions may indicate that even elderly population with relatively simple medication regimens, still need to be supported with their medication management. Of note, there are some factors that could have contributed to our findings but were not addressed by the MRCI score itself. Those may include socioeconomic and environmental factors. For instance, factors like having a competent care giver which can be either a vigilant family member or a licensed nurse practitioner who usually has adequate health literacy [37]. Nonetheless, it is unclear whether this applies to our study population since the majority of our patient are Qatari who are eligible to receive support from the government which often includes licensed nurses. Additionally, the setting in which the study was done may have contributed to the results as well. In Qatar, the elderly population with multiple comorbidities are serviced by the Home Health Care (HHC) service which provides multidisciplinary care in the community. Additionally, in our population, clinical pharmacy services are prominent upon discharge delivering the education needed to ensure safe and effective medications use. The number of co-morbidities that elderly HF patients usually have is another factor that was not addressed by MRCI tool and can directly impact clinical outcomes. Finally, the high-risk medications, as it was found that some medications are highly associated with hospital re-admissions in elderly population compared to other medications [38].

### Strengths

To the best of our knowledge, this is the first study in Qatar and the Middle East that investigated the relationship between MRCI score and important clinical outcomes in elderly patients with HF. We had used a validated tool, i.e., MRCI, calculated the sample size based on earlier and previously published research, and manually retrieved data from electronic health records by four experienced pharmacists to reduce likelihood of errors.

### Limitations

The study’s limitations include its retrospective design and observational nature, which precluded the examination of crucial factors influencing rehospitalization and medication adherence, such as patient cognition, dependence levels, caregiver availability, caregiver burden, and other geriatric syndromes. The patient recruitment from an advanced HF clinic, known for high-quality care, and the exclusion of emergency department visits may have positively influenced the clinical outcomes. Potential biases, such as information bias from manual MRCI score calculation and lack of interrater reliability testing, as well as performance bias from differential care or treatment, could impact rehospitalization or other outcomes. Moreover, the sample size might be insufficient for multivariate analysis in observational studies.

Future research should investigate the effects of dedicated homecare health services for the elderly upon discharge on hospitalization and readmission rates, as well as the role of various socioeconomic factors in elderly care.

## Conclusion

Polypharmacy and complex medication regimens are frequently observed in elderly heart failure (HF) patients. Despite this prevalence, our study found that medication regimen complexity, as measured by the MRCI, is not associated with hospital readmissions or mortality. Consequently, relying on MRCI as a predictor for rehospitalization in elderly HF patients may not be advantageous. It is important to consider other factors that could contribute to readmissions and mortality in this population, such as patient cognition, caregiver availability, and social support. By focusing on these factors and addressing the limitations of our study, future research may yield more accurate predictors and help improve care for elderly HF patients.

## Electronic supplementary material

Below is the link to the electronic supplementary material.

Supplementary Material 1

## Data Availability

All data generated during this study are included in this published article.
