# Supplementary figures and images for: Implications of the medication regimen complexity index score on hospital readmissions in elderly patients with heart failure: a retrospective cohort study
Source: BMC Geriatr. 2023 Jun 19;23:377. doi: 10.1186/s12877-023-04062-2 (PMC10278269; doi:10.1186/s12877-023-04062-2)

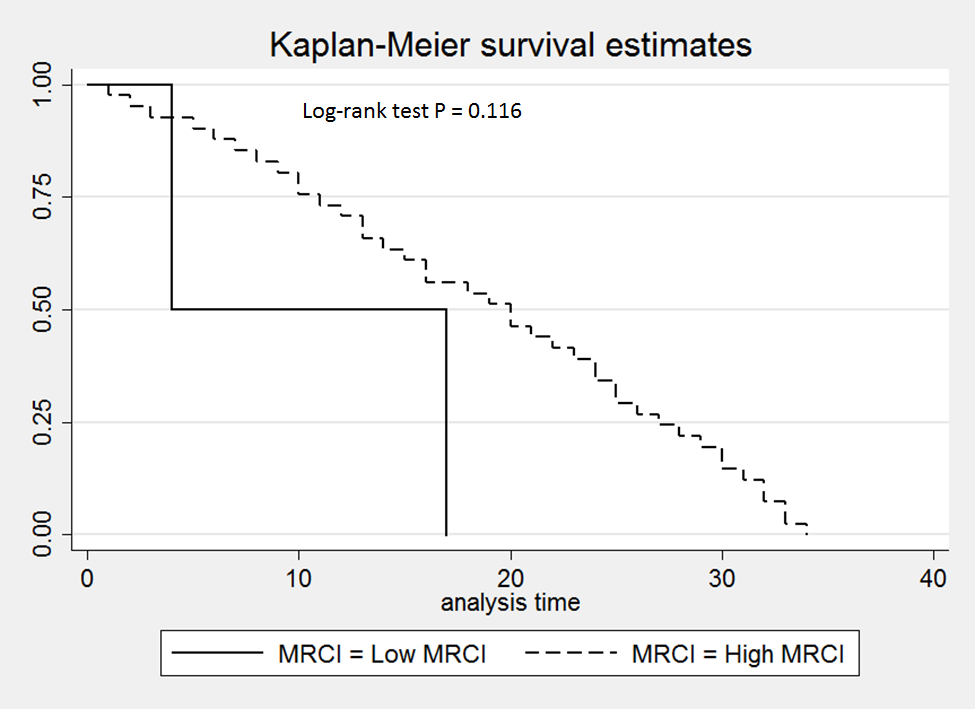


**Supplementary** **Figure 1: Kaplan-Meier survival curve**

Supplement: Supplementary file 1 — Supplementary Material 1 [file 12877_2023_4062_MOESM1_ESM.docx]
